# Supplementary material for: Detailed observations reveal the genesis and dynamics of destructive debris-flow surges
Source: Commun Earth Environ. 2025 Jul 16;6(1):556. doi: 10.1038/s43247-025-02488-7 (PMC12267052; doi:10.1038/s43247-025-02488-7)
Supplement: Supplementary file 2 — Supplementary Information [file 43247_2025_2488_MOESM2_ESM.pdf]

Detailed observations reveal the genesis and dynamics of destructive debris-flow surges

Supplementary Information

By: J. Aaron[1,2*], J. Langham[3], R. Spielmann[1,2], J. Hirschberg[1,2], B. McArdell[2], S. Boss[2], C.G. Johnson[3], J.M.N.T. Gray[3]

* Corresponding Author: jordan.aaron@eaps.ethz.ch

[1] Geological Institute, ETH Zurich, Zurich, Switzerland

[2] Swiss Federal Institute for Forest, Snow and Landscape Research (WSL), Birmensdorf, Switzerland

[3] Department of Mathematics and Manchester Centre for Nonlinear Dynamics, University of Manchester, Oxford Road, Manchester M13 9PL, UK

Supplementary Note 1: Flow Front and Surge Wave Propagation

The arrival of the flow front at the three measurement stations is shown in Supplementary Videos 1, 2 and 3. Surge waves at the two downstream measurements stations are shown on Supplementary Videos 4 and 5. A surge crest travelling faster than the material velocity is visible in Supplementary Video 6. Supplementary videos 7 and 8 show compression at the front of surge waves, indicating they are not merely surface phenomenon. Supplementary video 9 shows an animation of our simulation. It should be noted that the timestamps of the debris-flow videos is in UTC time, whereas local time is used in the manuscript.

Supplementary Note 2: Measurement of Velocity and Depth

As mentioned in the main text, we derive dense surface velocity fields for the measured channel reach, and use these to derive width-averaged velocities at a series of cross sections. We process these cross-section data to derive width-averaged depths, as described in the main text. Example flow velocity fields, the section lines used, and an instantaneous cross-section are shown for Gazoduc in Figure S 1, CD27 in Figure S 4 and CD 29 in Figure S 6. It should be noted that the flow velocity fields were filtered to remove outlier vectors, which leads to different spatial extents between the velocity field and measured flow surface. We further show the instantaneous depths across the representative sections in Figure S 2, Figure S 5 and Figure S 7.

Gazoduc

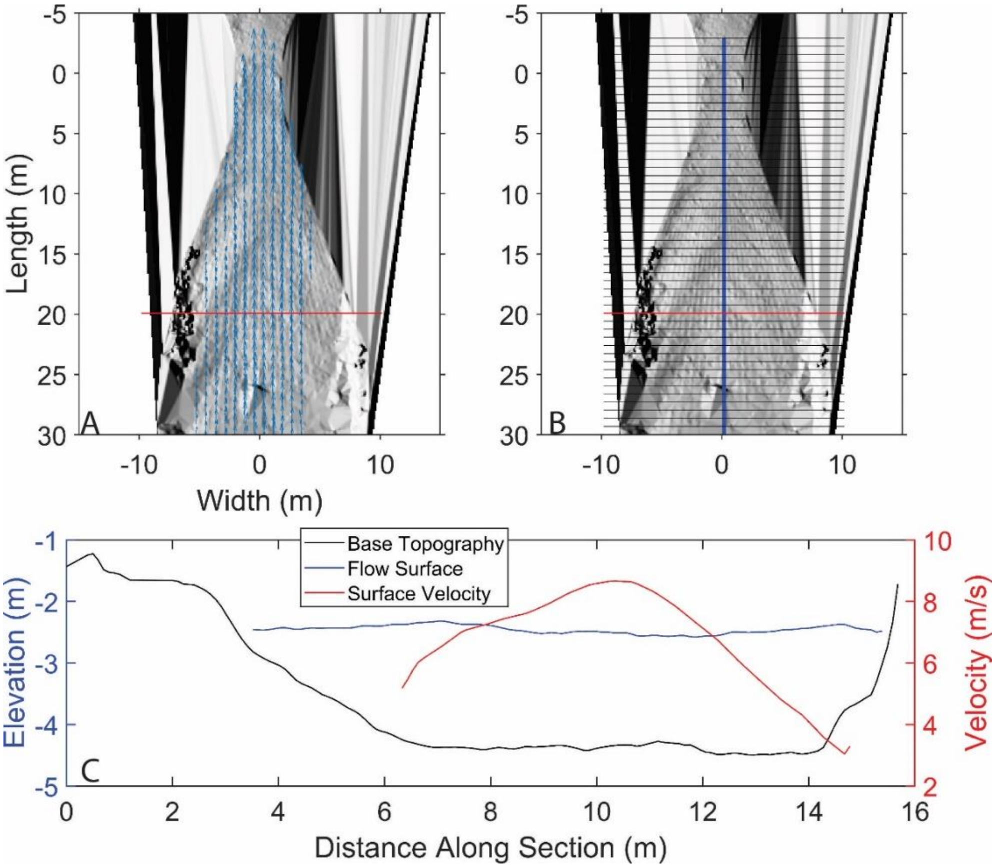

Figure S 1: Example data from Gazoduc showing how the width- averaged depth and velocity were estimated. A) Flow surface velocity vector field measured at 11:26:23 on June 5th, 2022. The red line shows the representative cross section used. The length is distance upstream of the sensor, and width is distance across the channel. B) cross sections where flow properties were derived, with the red cross section showing the representative cross section. The thick blue line shows the longitudinal cross-section used to align the lateral cross-sections. The background image in A) and B) is a hillshade projection of the flow surface. C) Data from the representative cross section, showing top surface and velocity measured at 11:26:23 on June 5th, 2022, as well as the base topography used. It should be noted that the data gets filtered outside the measured channel (x ≳ 15).

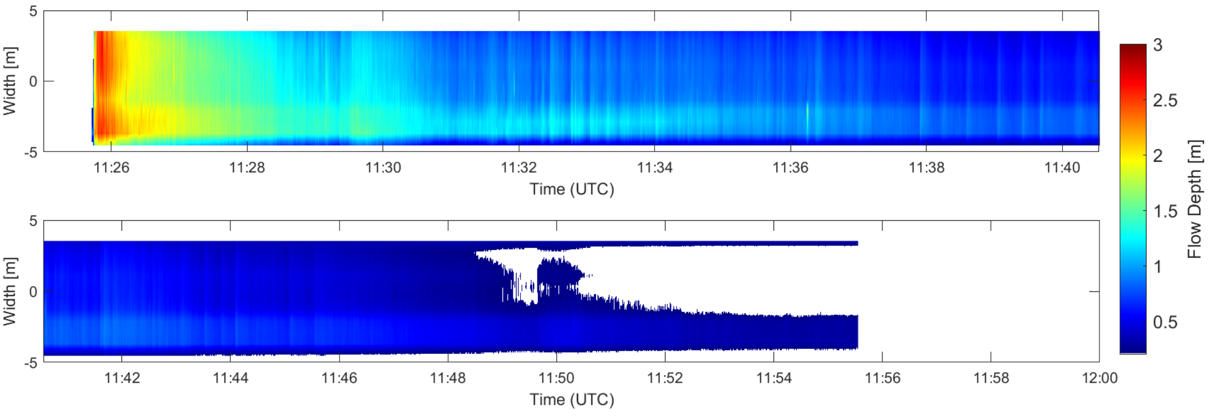

Figure S 2:Instantaneous depth through time at the representative cross section used for Gazoduc (red on Figure S 1).

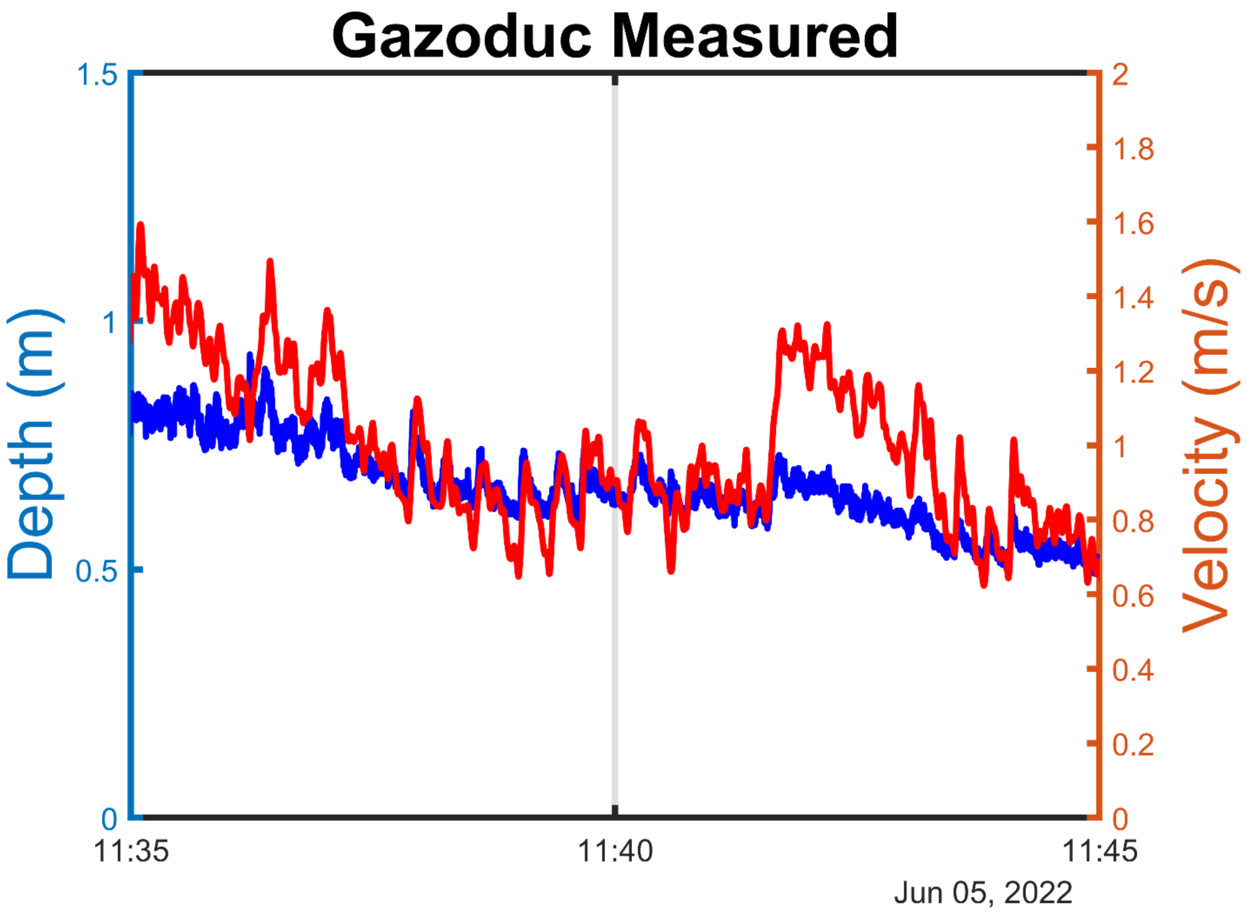

Figure S 3: Zoom in of Gazoduc velocity and depth timeseries showing surge development once the input flux has waned.

Check Dam 27

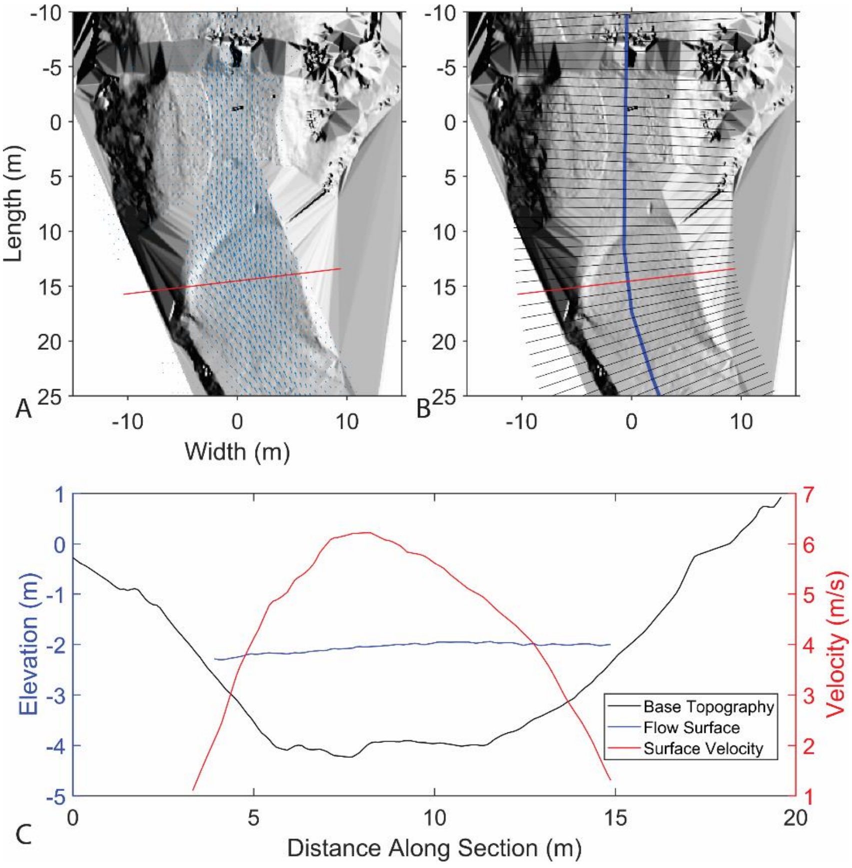

Figure S 4: Example data from CD 27. A) Flow surface velocity vector field, extracted from the measurements at 11:32:31 on June 5th, 2022, with the representative cross section highlighted in red. Length and width are defined in the caption for Figure S 1. B) All cross sections where flow properties were derived (representative cross section in red, longitudinal cross-section for alignment in blue). The background image in A) and B) is a hillshade projection of the instantaneous flow topography. C) Instantaneous data from the representative cross section measured at 11:32:31 on June 5th, 2022. The base topography is also shown.

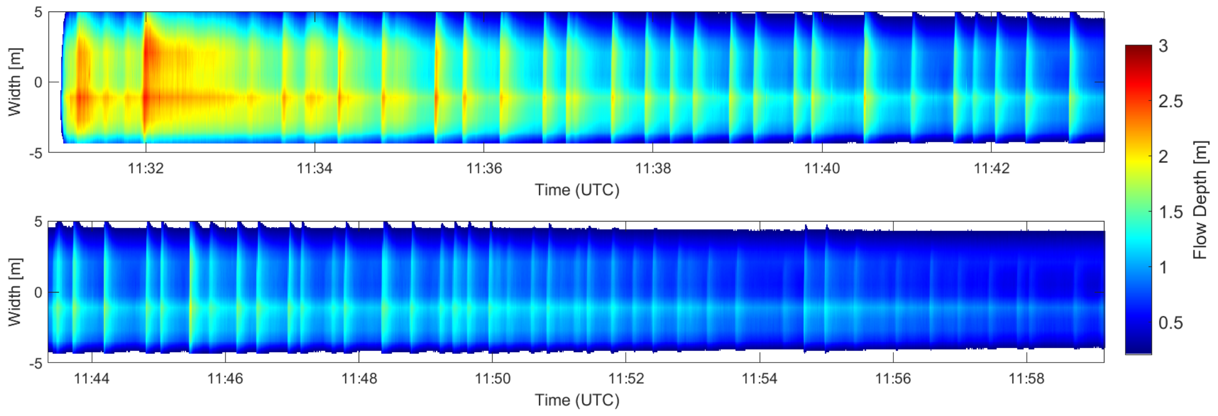

Figure S 5:Depth through time along the CD 27 representative cross section (red on Figure S 4).

Check Dam 29

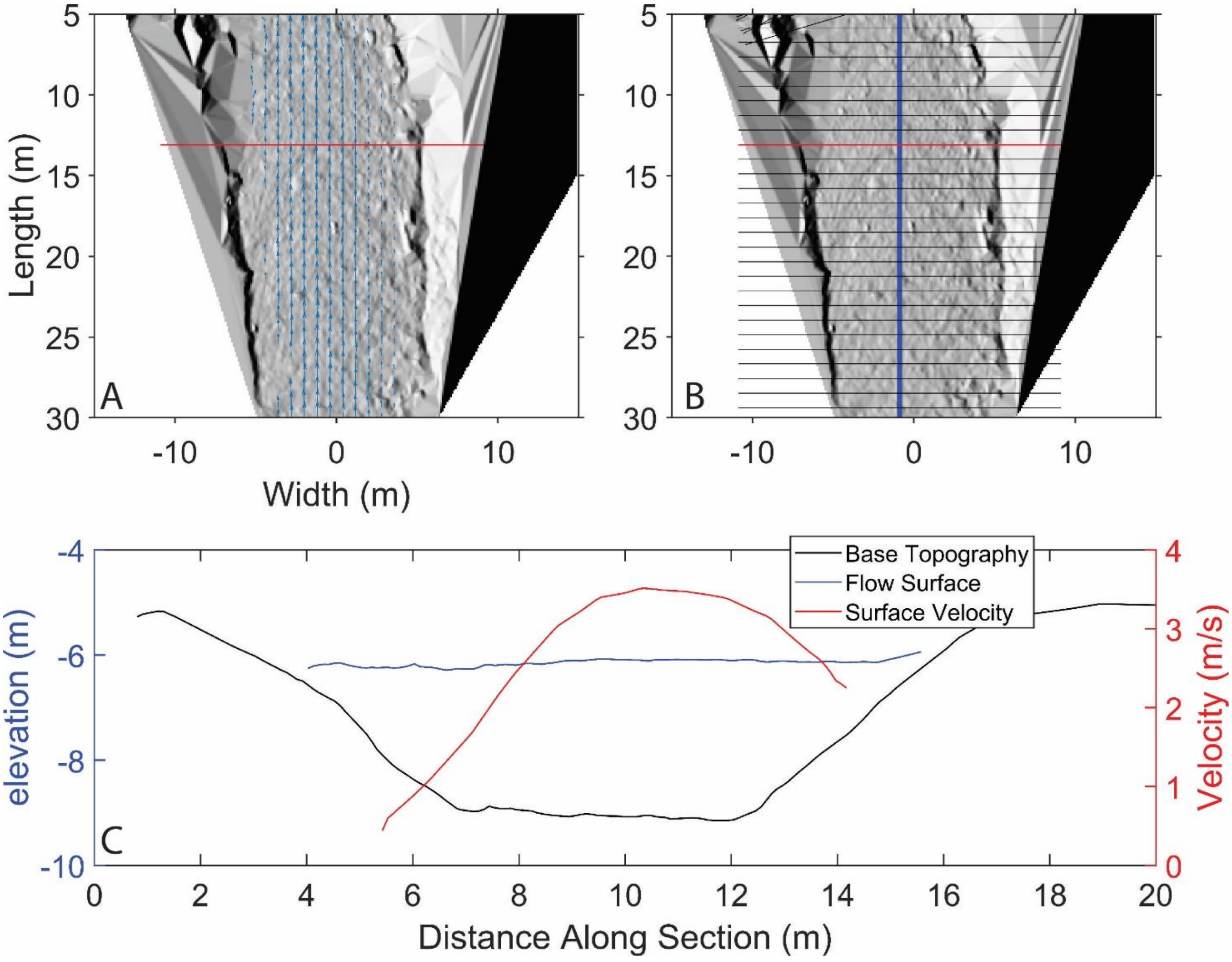

Figure S 6: CD29 flow surface velocity field measured on June 5th, 2022 at 11:34:14 and representative cross-section (A), with length and width defined in Figure S 1, evaluated cross sections (B) and (C) example data from the representative cross section (red on A and B).

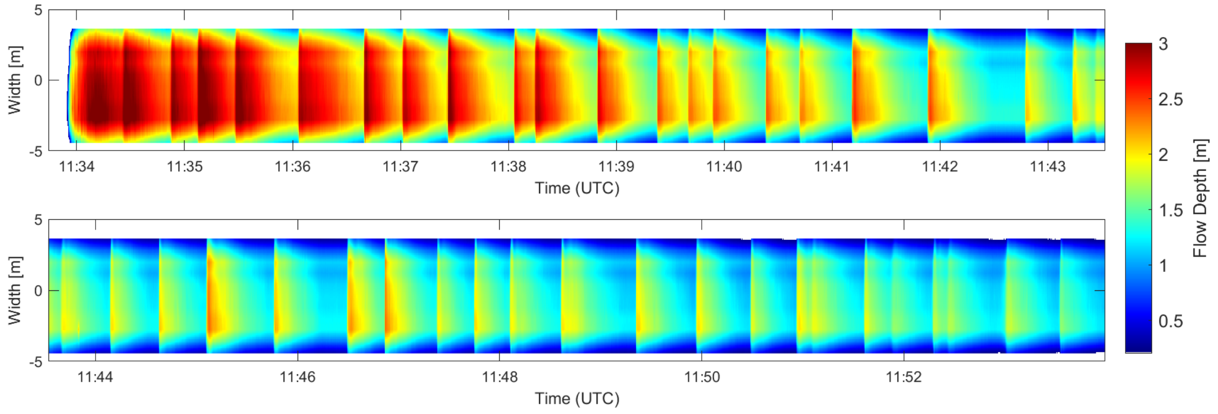

Figure S 7: Depth through time along the CD 29 representative cross section (red on Figure S 7).

Supplementary Method 1: Basal Friction Inversion

The definitions of the variables used in the basal friction inversion process are shown on Figure S 8.

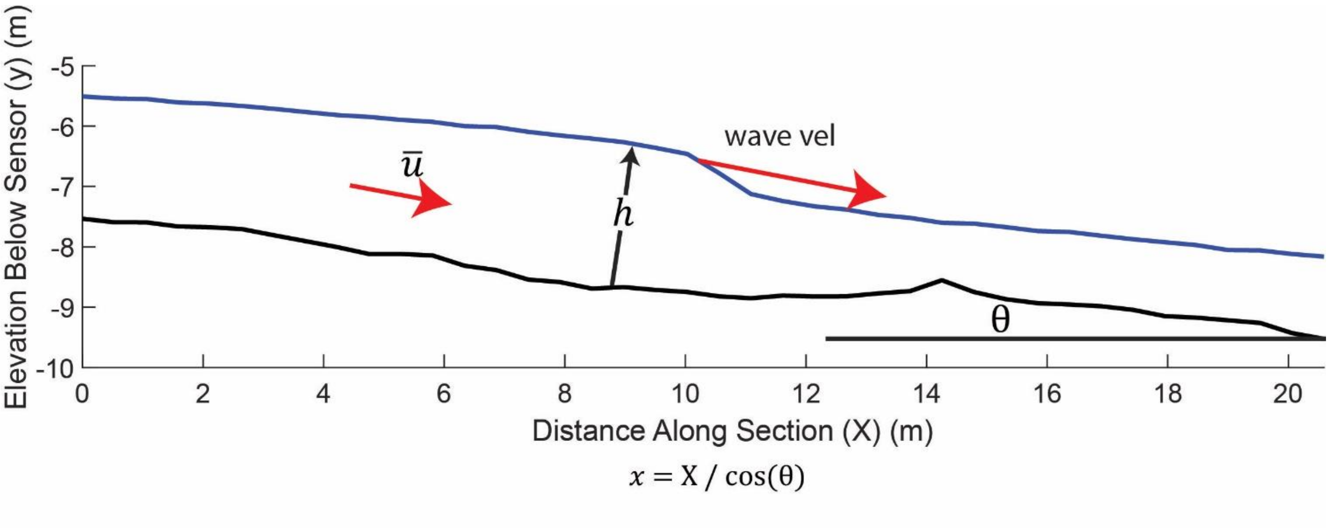

Figure S 8: Explanation of variables used in LiDAR data inversion.

We validated the basal friction inversion by comparing the estimated friction to the ratio of shear and normal force measured at a large force plate located at CD 29 [1]. We show two comparisons of the inverted data to these measurements on Figure S 9 and Figure S 10. Figure S 9 shows the force plate measurements directly compared to the output of the inversion procedure. There is an offset between the two, however the timing and magnitude of the basal resistance drops (which are induced by the passage of surge waves) matches well across the two datasets. The force plate is located at the brink of the check dam, and is therefore affected by the hydraulic drawdown that occurs at this location. On Figure S 10 we attempted to correct for this by subtracting the term corresponding to the depth gradients from the inverted friction coefficient ($\frac{\partial h}{\partial x}$ on Eqn [7] of the main text). As can be seen, this removes the offset and the two timeseries match well.

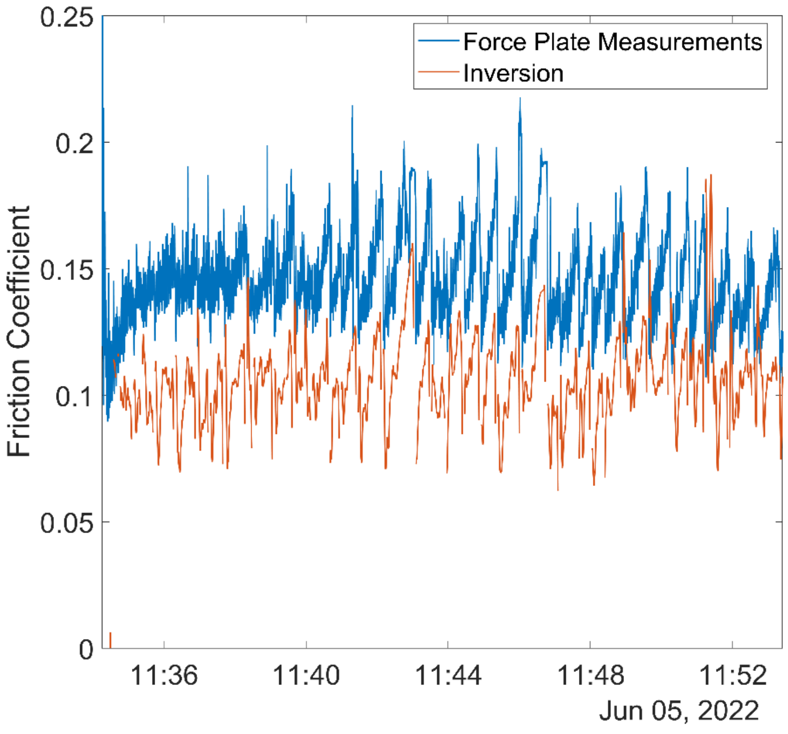

Figure S 9: Comparison of force plate measurements to inverted friction, with no correction made for the presence of a free overfall.

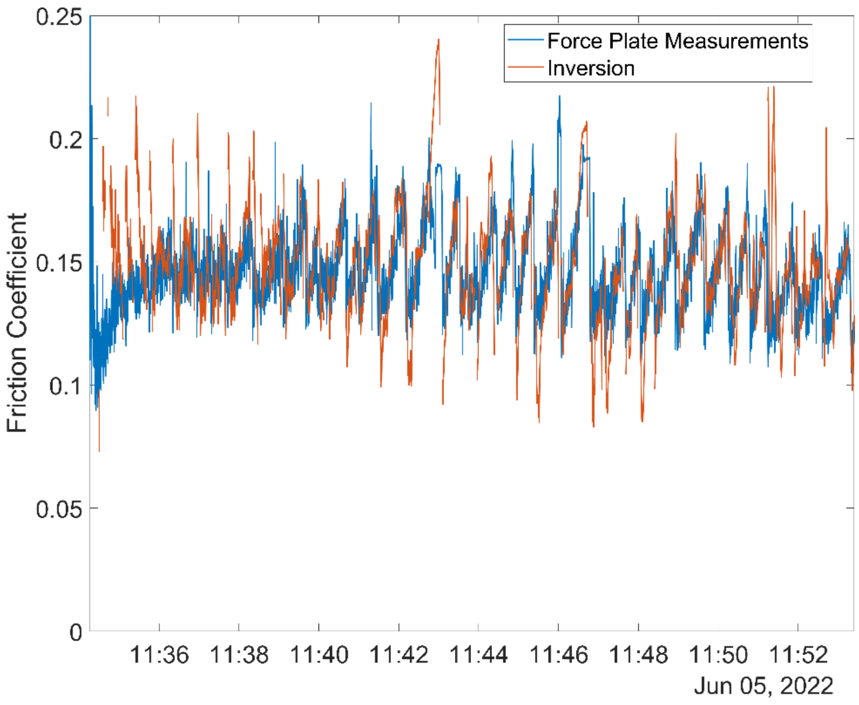

Figure S 10: Corrected comparison of inversion and force plate data, with presence of a free overfall removed

Supplementary Method 2: Numerical Model Parameter Selection

The parameters used in our numerical model do not directly correspond to quantities that are measurable in the field, or obtainable from current experimental data. Therefore, Eq. [9] in the main text may be viewed as a phenomenological construction that is flexible enough to approximate the inverted friction values and extend them to a plausible model friction function valid for all $Fr, h > 0$. It is possible to devise many different sets of parameters to those in Table 1 that approximate the friction values constrained by observations. This allows for a wide range of parameter choices that can quantitatively reproduce the essential dynamics of the instability and wave growth discussed in the main text, as shown in Figure S 11.

Nevertheless, there are some useful theoretical considerations that can be used to constrain the selection of parameters, which we detail below. Since the purpose of the simulation in this paper is to validate the reconstructed friction values and establish the relevance of the underlying roll wave instability, we did not perform extensive parameter fitting beyond this. Instead, we conducted several simulations (of which the Figure S 11 cases form a subset) to verify the robustness of our conclusions to variations in the parameters and chose to present the result with the Table 1 values as a representative example.

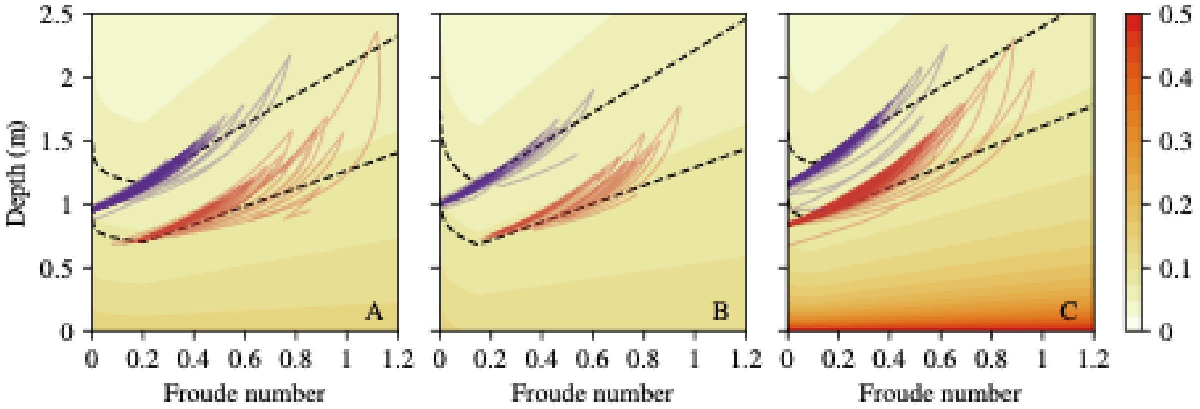

Figure S 11: Robustness of numerical results to variations in the frictional parameters. Each panel shows contours of friction, simulated Fr versus h at CD 27 (red) and CD 29 (purple), and the steady balances of Eq. [S1] with friction set by Eq. [9] for $\theta = 4.5°$ (lower dashed lines) and $\theta =$ $3.5°$(upper dashed lines). Panel A is the simulation presented in the main text, using the parameters

from Table 1, while panels B and C use the following modified values: (B) $\mu_1 = -0.025, \mu_2 = 0.12, \beta = 0.5, \beta_\star = 0.15, \kappa = 0.2$*; (C)* $\mu_1 = 0.02, \mu_2 = 0.5, \beta = 8, \beta_\star = 0.1, \kappa = 0.01, \Gamma = 1$*. Note that the three cases lead to friction functions that are relatively close to the observed friction where data is available, but diverge from one another outside this region. The colorbar shows the friction coefficient.*

Three parameters in the friction law, μ_3, L, κ control the behaviour of the friction law near the onset of deposition ($Fr < \beta_\star$) and do not play a role in the roll wave instability. The value of μ_3 and the length scale L dictate the range of uniform flows that may exist as static deposits at a given slope angle. Specifically, an initially uniform static deposit of depth h can only remain stationary if $\mu_{start}(h) > \tan\theta$, which rearranges to give

$$h < L\left(\frac{\mu_2 - \mu_1}{\tan\theta - \mu_3} - 1\right).$$

Since we know that static deposits did form at CD 29, this places a constraint on μ_3 and L (given μ_1 and μ_2, which are dependent on considerations below). However, without additional data, such as the yield strength of the debris, this does not fully determine these parameters. Therefore, we chose some physically plausible values and verified that simulations are qualitatively insensitive to reasonable adjustments of these selections. (For reference, using our chosen values in the inequality above implies that uniform static layers can form for $h < 1.24$m at CD 27 and $h < 2.16$m at CD 29.)

The variable κ controls the behaviour of the low Fr frictional regime, which governs the onset of flow arrest and is only accessed intermittently by the waves that form deposits at their tails. A higher value of κ causes deposits to form more rapidly whenever they are triggered and therefore influences the shape of erosion-deposition waves. We set it to a value that produced waves with reasonable qualitative resemblance to the observed profiles at CD 29. Roll waves transition into erosion-deposition waves when their tails drop below $Fr < \beta_\star$. Higher values of $\beta_\star$ somewhat enhance the phenomenological distinction between the two wave classes by allowing erosion-deposition waves to form more readily. Our chosen value ($\beta_\star = 0.2$) is large enough to promote the formation of clear

erosion-deposition waves (as observed in the field), but not so large that it interferes with the roll wave instability that occurs at higher Fr.

The remaining four parameters, determine the friction values in the dynamic regime ($Fr > \beta_\star$), within which the initiation and development of roll waves are observed in the field data. Roll waves emerge from a linear instability of steady uniform flow. Under steady uniform conditions, gravitational forcing is exactly balanced by frictional resistance:

$$\mu_b(Fr, h) = \tan\theta \qquad [\ S1\ ]$$

which, for each slope angle θ, defines a curve in (Fr, h) space. When $Fr > \beta_\star$ this may be rearranged to give the linear relationship

$$h = \left(\frac{L}{\beta} \cdot \frac{\mu_2 - \tan\theta}{\tan\theta - \mu_1}\right)(Fr + \Gamma), \qquad [\ S2\ ]$$

Since, h and Fr are both positive, $L/\beta > 0$, and $\tan^{-1}\mu_1$ and $\tan^{-1}\mu_2$ are respectively interpreted as minimum and maximum slope angles upon which uniform steady flows can exist. Therefore, $0 < \tan^{-1}\mu_1 < \theta < \tan^{-1}\mu_2$ (although even permitting $\mu_1 < 0$ can produce a reasonable fit to the reconstructed friction, as demonstrated in Figure S 11B).

To restrict these parameters further, we note that in their idealised description as steady, stable travelling wave solutions to Eqs. [4] and [5] (main text), roll waves are mathematically constrained to contain flow with the values (Fr_0, h_0) at a 'critical point' somewhere along their length, where Fr_0 and h_0 are the Froude number and depth of the corresponding uniform flow that gave rise to them via linear instability[2]. In other words, at a given slope angle, all roll waves must pass through the line defined in Eq. [S2] and the location of this intersection is contingent on the upstream flux (which controls Fr_0, h_0). This can be seen in the simulation data of Figure S 11, which are 'pinned' to the black dashed lines. The field data in Figure 4 (main text) are clearly separated into quantitatively different regimes, consistent with the different mean slope angles at the two downstream measurement stations. Therefore, we selected μ_1, μ_2, β, and Γ so that the Eq. [S2] lines pass through the field data at CD 27 ($\theta = 4.5°$) and CD 29 ($\theta = 3.5°$). Note that given a systematic procedure for

deciding on optimal intersection points, wave data at three different slope angles would be required to uniquely determine μ_1, μ_2, β and Γ. Therefore, we choose illustrative values, which lead to good agreement with the inferred field values of friction, within the available data ranges, as demonstrated in Figure 4 (main text).

Finally, we performed a set of simulations that don't vary the slope angle. The results of these, shown on Figure S12, demonstrate that a slope angle change is necessary to preproduce the data separation observed in the field data.

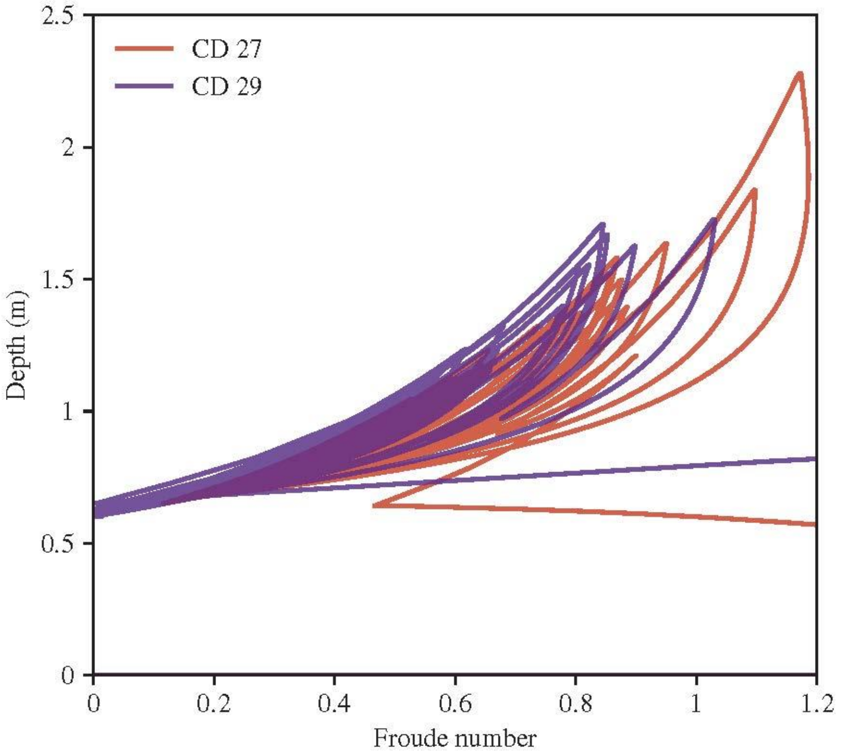

Figure S 12: Parameter space accessed by the simulation when a constant slope is used. As can be seen, the CD 27 and CD 29 data do not clearly separate, as observed in Figure 4 of the main text.

Supplementary Discussion 1: Simulated Discharge and Basal Friction

The simulated values of discharge and basal friction can be seen on Figure S 12, and compared with the measured results on Figure 3 of the main text. As can be seen, the overall agreement is good, however front discharge is underestimated by the model at CD 27 and CD 29, and basal friction slightly overestimated at CD 29.

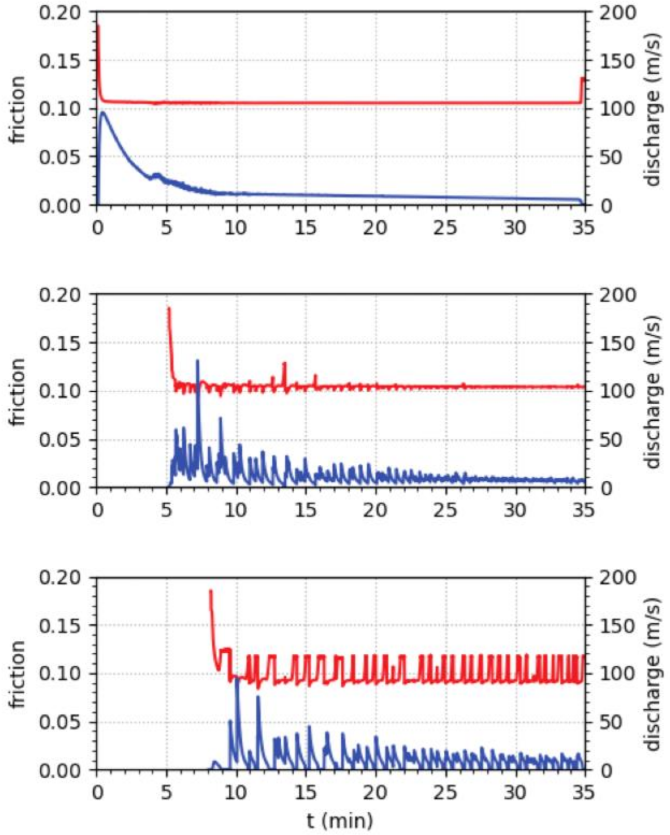

Figure S 13: Simulated discharge and basal friction.

Supplementary References

1. McArdell, B. W., Bartelt, P. & Kowalski, J. Field observations of basal forces and fluid pore pressure in a debris flow. *Geophys. Res. Lett.* **34**, 2–5 (2007).
2. Dressler, R. F. Mathematical solution of the problem of roll-waves in inclined open channels. *Commun. Pure Appl. Math.* **2**, 149–194 (1949).
