# Supplementary material for: Detailed observations reveal the genesis and dynamics of destructive debris-flow surges
Source: Commun Earth Environ. 2025 Jul 16;6(1):556. doi: 10.1038/s43247-025-02488-7 (PMC12267052; doi:10.1038/s43247-025-02488-7)
Supplement: Supplementary file 3 — Description of Additional Supplementary files [file 43247_2025_2488_MOESM3_ESM.pdf]

Description of Additional Supplementary files

File name: Supplementary Video S1

Description: Source data for Arrival of the flow front at Gazoduc.

File name: Supplementary Video S2

Description: Arrival of the flow front at CD 27.

File name: Supplementary Video S3

Description: Arrival of the flow front at CD 29.

File name: Supplementary Video S4

Description: Example surge wave at CD 27.

File name: Supplementary Video S5

Description: Example surge wave at CD 29.

File name: Supplementary Video S6

Description: Video showing a surge wave crest traveling faster than surface particles.

File name: Supplementary Video S7

Description: Surge wave at CD 27 which shows compression of the surge wave front, which indicates that the waves are more than surface phenomenon.

File name: Supplementary Video S8

Description: Surge wave at CD 29 which shows compression of the surge wave front, which indicates that the waves are more than surface phenomenon.

File name: Supplementary Video S9

Description: Animation of the numerical modelling results.
